# Estimation of ground reaction forces and ankle moment with multiple, low-cost sensors

**DOI:** 10.1186/s12984-015-0081-x

**Published:** 2015-10-14

**Authors:** Daniel A. Jacobs, Daniel P. Ferris

**Affiliations:** School of Kinesiology, University of Michigan, 401 Washtenaw Ave CCRB, Ann Arbor, MI USA

**Keywords:** Locomotion, Gait, Biomechanical analysis, Plantar pressure insole, Ground contact, Sensor fusion

## Abstract

**Background:**

Wearable sensor systems can provide data for at-home gait analyses and input to controllers for rehabilitation devices but they often have reduced estimation accuracy compared to laboratory systems. The goal of this study is to evaluate a portable, low-cost system for measuring ground reaction forces and ankle joint torques in treadmill walking and calf raises.

**Methods:**

To estimate the ground reaction forces and ankle joint torques, we developed a custom instrumented insole and a tissue force sensor. Six healthy subjects completed a collection of movements (calf raises, 1.0 m/s walking, and 1.5 m/s walking) on two separate days. We trained artificial neural networks on the study data and compared the estimates to a multi-camera motion system and an instrumented treadmill. We evaluated the relative strength of each sensor by testing each sensor’s ability to predict the ankle joint torque calculated from a reference inverse kinematics algorithm. We assessed model accuracy through root mean squared error and normalized root mean square error. We hypothesized that the estimation of the models would have normalized root mean square error measures less than 10 %.

**Results:**

For walking at 1.0 and walking at 1.5 m/s, the single-task, intra-day and multi-task, intra-day predictions had normalized root mean square error less than 10 % for all three force components and both center of pressure components. For the calf raise task, the single-task, intra-day and multi-task, intra-day predictions had normalized root mean square error less than 10 % for only the anterior-posterior center of pressure. The multi-task, intra-day model had similar predictions to the single-task, intra-day model. The normalized root mean square error of predictions from the insole sensor alone were less than 10 % for walking at 1.0 m/s and 1.5 m/s. No sensor was sufficient for the calf raise task. The combination of the insole sensor and the tendon sensor had lower normalized root mean square error than the individual sensors for all three tasks.

**Conclusions:**

The proposed sensor system provided accurate estimates for five of the six components of the ground reaction kinetics during walking at 1.0 and 1.5 m/s and one of the six components during the calf raise task. The normalized root mean square error of the predictions of the ground reaction forces were similar to published studies using commercial devices. The proposed system of low-cost sensors can provide useful estimations of ankle joint torque for both walking and calf raises for future studies in mobile gait analysis.

## Background

Increasing health care costs and the need for more convenient and cost-effective patient care are driving investigations into research and development of mobile health monitoring systems. Advances in wearable technologies, as one subgroup of mobile health monitoring technologies, can enable more affordable and accessible health care by developing low-cost, unobtrusive measurement devices that can provide real-time feedback to patients and health care providers on the patient’s health in their every day lives [1, 2]. The timely measurement and communication between wearable technologies, patients, and health care providers could have significant effects on the quality of life of patients by helping drive health care from treatment to prevention [3].

Estimating the ground reaction forces and joint moments of humans in the real world could have substantial clinical impact by providing assessments of pathological gait, fall detection in the elderly, and biofeedback data for home interventions. Currently, the gold-standard of clinical gait analysis using motion capture and force plates can estimate ground reaction forces and joint moments without invasive sensors. However, the viability of gait analysis is restricted due to their size, cost, and laboratory size. The aim of this study was to test the ability of multiple sensors to provide low-cost measurements of ground reaction forces and ankle joint moments.

Many wearable sensors have difficulty providing robust estimations in the presence of substantial movement variability. Studies on wearable sensors that only investigate overground and treadmill walking do not deal with the amount of variability that happens in daily life [4–8]. In some cases, the investigated sensor is tested only on the stance phase of walking [9, 10]. Investigating only walking leads to overestimation of sensor accuracy due to the fact that the passive dynamics of walking lead to stereotypical patterns that are easier to predict. Not including swing phase data in estimation also reduces variability in the data set because it assumes that nonlinear transition between stance and swing is predicted perfectly despite sensor noise.

Investigating non-stereotypical tasks can lead to more accuracy assessments of wearable technology. One non-stereotypical task that could be included in performance testing of wearable technologies is the calf raise. Biomechanically, calf raises are interesting because the major functional behavior is balancing rather than forward propulsion like in walking. The profiles of ground reaction force and center of pressure during a balancing task have larger variance than a stereotypical task which makes accurate prediction more challenging. Clinically, calf-raises are often elements of lower limb rehabilitation protocols following Achilles tendon and anterior cruciate ligament injury [11, 12] and training protocols for improving balance and gait stability [13].

We evaluated a system consisting of two custom, low-cost sensors: a custom plantar pressure insole and a non-invasive tendon load cell. By fusing multiple sensors, the estimation accuracy of the sensor system could be reduced enough to create an acceptable, generalizable model that is robust and repeatable across different tasks. Several groups have demonstrated that plantar pressure insoles provide sufficient data to estimate ground reaction forces (GRF), center of pressure (COP), and ankle joint torques (AJT) [5, 6, 8] yet insole performance on non-stereotypical tasks is unclear. Tendon sensors, such as tendon buckles [14, 15], non-invasive strain sensors [16], and ultrasonic velocity measurements [17] have also provided useful data on muscle and tendon state but most sensors are bulky and require specialized equipment that is not mobile.

In addition to task variability, sensor characteristics, such as drift and creep, can negatively affect estimation with insoles [18–20]. Studies on insole sensor variability and repeatability have shown that current devices need calibration in order to perform adequately [21, 22] and that long term performance is still an issue [21, 23–26].

In this study, we quantified the predictive ability of the sensors to estimate the ground reaction forces, center of pressure, and ankle joint torques during normal walking and calf-raises in healthy young adults. We collected steady state treadmill walking at two speeds and a set of five self-paced calf raises. To evaluate long-term performance, we collected trials on two different days without external calibration of the sensor or fitting. We built artificial neural network regression models to estimate the ground reaction forces, center of pressure, and ankle joint torques from the prototype sensor data and compared the predictions to the reference data from motion capture and instrumented force plates. To assess model accuracy, we performed a series of estimations on withheld data and calculated the mean performance measures across intra-day, inter-day, single-task, and multi-task groupings. We used the root mean square error (RMSE) and the normalized root mean square error (NRMSE) as measures of model performance.

## Methods

### Subjects

Six healthy subjects (2 Female, 4 Male) participated in this study: (mean ± std) age 24.5 ± 3.6 years, height 1.78 ± 0.07 m, leg length 0.94 ± 0.05 m, and mass 69.9 ±12.64 kg. Each collection day, the subjects performed three trials of walking at 1.0 m/s, walking at 1.5 m/s, and three sets of five calf raises. Each subject performed two collections spaced 40 ± 15 days apart.

### Ethics, consent and permissions

All subjects gave written informed consent in accordance with the Declaration of Helsinki. The Institutional Review Board of the University of Michigan (FWA #00004969) approved the study protocol.

### Data collection

We recorded marker data at 100 Hz with a 10 camera motion capture system (Vicon, Inc. USA). We collected ground contact forces at 1 kHz with an instrumented split-belt treadmill (Bertec Inc. USA). A data acquisition system (dSPACE GmhB, Germany) running Real-Time Workshop (Mathworks Inc., USA) captured the voltage signals from the insole and tendon sensors at 1 kHz. We filtered the ground contact forces and moments from the instrumented force plates and the voltage signals from the sensors with a low-pass, fourth order butterworth filter at 25 Hz. We synchronized the data acquisition system and the motion capture system by co-recording a manually-triggered square wave from a signal generator in both systems. The data streams were aligned by edge detection of the square wave in the post-processing routines.

In a few trials, an intermittent break in the electrical connection added artifact noise to the sensor data. The noise did not strongly contaminate the data and we did not filter the artifact or exclude any data from our analysis.

### Sensing hardware

During the study, each subject wore an orthopedic shoe with a custom insole insert to measure localized changes in plantar pressure. Each insole contained eight custom neoprene bladders instrumented with miniature, amplified, temperature-compensated pressure sensors (Honeywell, Inc. USA, SSCDANN030PGAA5). The pneumatic bladders had a loading area of 25.4 × 25.4 × 6.35 mm and a wall thickness of 1.58 mm. The total cost of a pair of instrumented shoes was $800 (Sensors: $512, Bladders: $144, PCB $132, insole foam, glue, and misc: $10). The base weight of the shoe was 480.2 g and total weight of the sensors was 257.8 g which accounted for an increase of 54 percent over normal attire (Table 1).

**Table 1:** Mass values of a single side of sensor system. The total weight of the insole sensors, Achilles tendon sensors, and circuitry increased the base weight of the shoe by 54 %

Part	Mass
Pressure Sensors and Circuitry	137 g
Achilles Tendon Sensor	44 g
Bladder	10 g
Sensor Subtotal	258 g
One Shoe (US Men’s 10)	480 g
Total Weight	738.0 g

Each subject also wore a miniature beam load cell on the distal end of the tibia above the calcaneus to measure localized tissue forces around the Achilles tendon. The tendon sensor consisted of a thin film load cell (Strain Measurement Devices, Inc. USA, S100) connected to base aluminum block and a delrin tendon cup. The total cost for the pair of the tendon sensors was $340 (Sensors $330, misc aluminum and delrin $10).

**Fig. 1:**
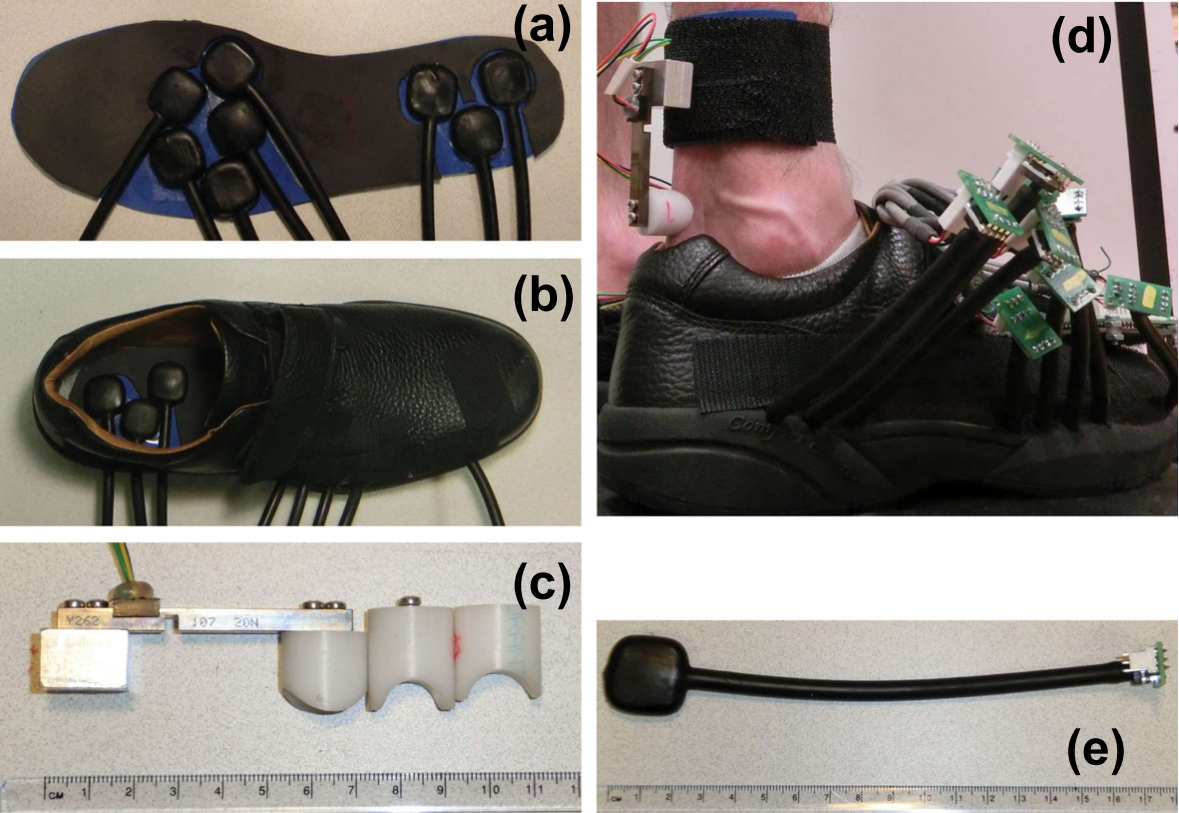
Hardware photos. The collage shows photos of **a** the insole and inlaid pressure sensors (top foam layer missing) **b** the insole embedded in the orthotic shoe, **c** a miniature beam load cell and the plastic tendon cups for the tendon sensor, **d** a subject wearing both of the sensors and **e** the custom pressure sensor and amplifier unit. The total cost of a pair of insoles was $800 and the total cost of the pair of load cells was $420. Both sensors were easily constructed, assembled, and disassembled without specialty tools

Both devices are simple to manufacture with standard laboratory tools and they can be adjusted for different users easily. Figure 1 shows several photos of the individual components and the full system worn by subject. Figure 1a and b show the insole (without the top comfort foam layer) with the bladders inserted. Figure 1e shows the bladder and custom printed circuit board for the pressure sensors. Figure 1c shows a tendon sensor along with two acetal resin tendon cups to assist in fitting different subjects. Figure 1d shows the complete system on a subject. We affixed the tendon sensor to the subject with double sided tape, then secured the entire sensor in flexible athletic tape.

### Analysis

We calculated the ground reaction forces (GRF), center of pressure (COP) and vertical torque in the laboratory coordinate system. The center of pressure and the vertical torque components of the ground reaction forces were set to zero when the vertical force measurement was less then 5 % of body weight.

We used a 23 degree of freedom musculoskeletal model (gait2354) in the open-source software OpenSim [27] for our analysis. Using the marker and treadmill data, we scaled the model and ran inverse kinematics and inverse dynamics algorithms to calculate the generalized coordinates and generalized forces of the model in each trial. We transformed the location of the center of pressure from the laboratory coordinate system to a foot fixed coordinate system with the origin in the calcaneus to normalize the subjects steps and remove the effect of treadmill position.

For the mean component profiles comparison, we normalized each subject’s step to gait cycle phase or task phase. We normalized the center of pressure locations as a percentage of the leg length (% L), forces as a percentage of body weight (% BW), and moments as a percentage of the product of body weight and leg length (% BWL). We estimated the leg length of the subject from the mean of the left and right greater trochanter z markers during the standing trials.

For the prediction of the GRF, we calculated the root mean square error (RMSE) and the normalized root mean square error (NRMSE). The NRMSE was calculated by dividing the RMSE by the range of the signal data in the reference signal from the motion capture and instrumented force plate data. For the prediction of the AJT, we also compared the relative strength of the individual sensors in the sensor set by calculating the accuracy for combinations of sensors. As a feature set, we compared the individual ankle angle estimated from the motion capture data, the voltage from the tendon sensor, and the vertical force and fore-aft center of pressure estimated from the insole sensor.

Although the true measure of accuracy for clinical validity is unknown, we set the accuracy cutoff at 10 % for this study to match the reported accuracy (5–28 %) of studies on walking that used commercial pressure and insole mats as those devices are utilized in clinics.

#### Regression models

We created a series of single-hidden layer, 10 node, feed-forward neural networks in Matlab. Each model was tested on data withheld from the training phase. We used a cross-fold validation scheme to create a series of models for each subject and calculated the mean model accuracy across the iterations [28].

Splitting data for regression models can be done supervised or unsupervised depending upon the experimental data [29]. We chose a supervised method where we split the training and test data into groups of intra-day and inter-day sets in order to ask interpretable questions, e.g., how accurate is the model likely to be when tested on subjects within a testing day or on a new testing day.

It is important to split the data into sections where the regions of prediction are equivalent to avoid local information and improve the bias properties of the predictors [29]. Splitting the data into intra-day and inter-day sets creates equivalent prediction areas because each set contains an equal number of cycles and the range and variance of the signal is similar. Splitting the data into randomized sections could result in an uneven split of the gait cycle which would have different range and variance properties which could bias the estimate of model accuracy.

From the data set, we created the following groups:

- Single-Task, Intra-Day
- Single-Task, Inter-Day
- Multi-Task, Intra-Day

**Fig. 2:**
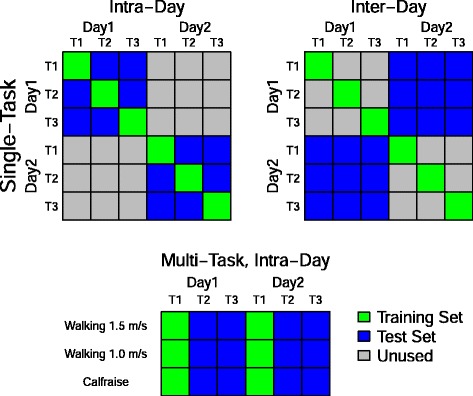
Analysis group illustration. On each collection day, we collected three trials of each task for a total of six trials. (*TOP*) In the single-task groups, we formed a six-by-six grid of training and test data sets for each task. The top three grids show a graphical representation of the three single-task groupings. The green squares represent the trial from which the training and cross validation data were taken and the blue squares represent the test sets which were averaged for the results. Note that union of the blue squares represents the complete grid with each set used once. (*BOTTOM*) For the multi-task group, we created a data set of eighteen trials from the three trials on both days for the three tasks. We created a training set using the first trials on both days for all three tasks and a test set from the second and third trials on both days

Our training and testing groups are represented visually in Fig. 2 in a grid representation. For the single-task sets, each row represents a set of tests, where the green boxes indicates the trial used for training and the blue boxes represent the trials used for testing.

For example, the single-task, intra-day set consisted of testing each trial on a set of withheld data consisting of the remaining two trials in that day e.g the model trained on the first trial was tested on the data from the second and third trials on that day and the model trained on the second trial was tested on the data from the first and third trials on that day.

In the single-task, intra-day, the grid coloring in Fig. 2 indicates that for each of the 6 models (green boxes) we produced 12 test performance measures (blue boxes) yielding a final performance metric that was the average of 120 values (10 subjects, 12 tests) for each kinetic component, task, and subject.

The multi-task, intra-day group, consisted of a single model trained on the first trial of each task from day 1 and day 2 and tested on the remaining two trials from each day (6 training trials and 12 testing trials). The final performance metric for each component was also the average of 120 values (10 subjects, 12 tests) for each kinetic component.

The neural network training algorithms further divided the input data into subgroups allocated as: 70 % training, 15 % validation, and 15 % test. To avoid over-fitting the data, the Matlab training algorithm halted the training when the performance decreased on the validation data set after an iteration. The output of each individual training on the remaining 15 % test data was discarded in favor of the output from the grid tests.

For each model, we calculated the root mean square error (RMSE) and the normalized root mean square error (NRMSE) on the withheld test data. For the final reported model performance, we calculated the mean RMSE and NRMSE across all subjects.

## Results

**Fig. 3:**
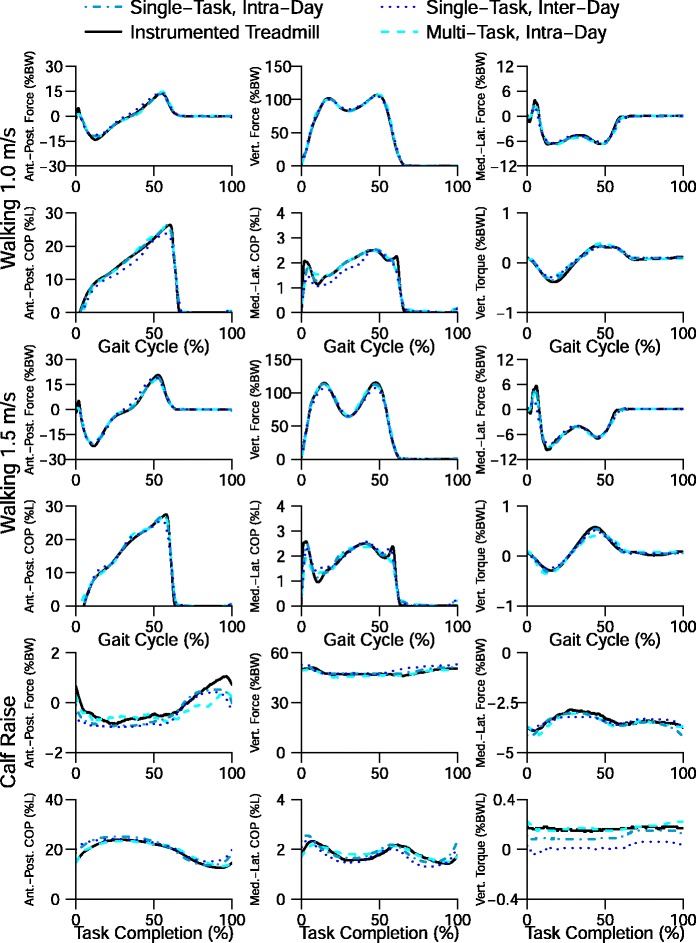
Ground reaction kinetics estimation. Mean normalized components of the ground reaction kinetics for walking at 1.5 m/s and calf raises for four analysis groups and the instrumented treadmill. The mean walking prediction is very accurate for all of the groups except for the Single-Task, Inter-day group which has an error around 40 % of the gait cycle. The error for the calf raise trial is noticeably larger than that of the walking trials. The difference in mean predicted curve is also larger for the calf raise trials. The increase in error is likely due to having fewer sensors available when subjects are on the balls of their feet. Component Names: Anterior-Posterior Force (*F*_*ap*_), Vertical Force (*F*_*v*_), Medial-Lateral Force (*F*_*ml*_), Anterior-Posterior Center of Pressure (*C* *O* *P*_*ap*_), Medial-Lateral Center of Pressure (*C* *O* *P*_*ml*_), Vertical Torque (*T*_*v*_)

For walking at 1.0 m/s, 1.5 m/s and calf raises, the differences between the mean ground reaction kinetics for the reference (solid black line) and estimated (blue lines) were small (Fig. 3). The differences between the reference and estimated curves are greatest for the single-task, inter-day subgroup.

**Fig. 4:**
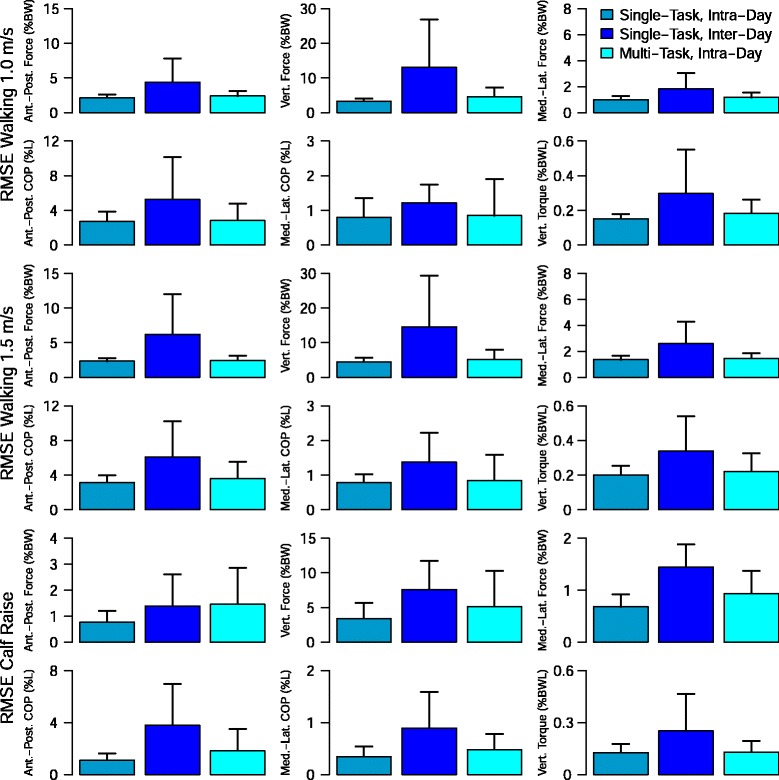
Ground reaction kinetics estimation statistics. Normalized root mean square error (NRMSE) for the three analysis groups on each of the three tasks. The quality of fit is stronger for the single-trial, intra-day groups than for the single-trial, inter-day group. The multi-trial, inter-day group shows that training on a large multi-task and trial dataset can reduce the inter-day error. Component Names: Anterior-Posterior Force (*F*_*ap*_), Vertical Force (*F*_*v*_), Medial-Lateral Force (*F*_*ml*_), Anterior-Posterior Center of Pressure (*C* *O* *P*_*ap*_), Medial-Lateral Center of Pressure (*C* *O* *P*_*ml*_), Vertical Torque (*T*_*v*_)

The normalized root mean square error for the single-task, inter-day group was than the single-task intra-day group for all components and tasks (Fig. 4 and Table 2). The multi-task, intra-day group had mean normalized root mean square error lower than the single-task, inter-day group for all components and tasks except for the anterior-posterior force component of the calf raise task.

**Table 2:** Fit metrics for the ground reaction kinetic estimation

	*F*_*ap*_		*F*_*v*_		*F*_*ml*_	
Walking 1.0 m/s	RMSE (% BW)	NRMSE (%)	RMSE (% BW)	NRMSE (%)	RMSE (% BW)	NRMSE (%)
Single-Task, Intra-Day	2.13	6.21	3.33	2.93	1.00	5.82
Single-Task, Inter-Day	4.40	13.07	13.06	11.48	1.85	10.97
Multi-Task, Intra-Day	2.39	6.95	4.72	4.15	1.16	6.79
	*C* *O* *P*_*ap*_		*C* *O* *P*_*ml*_		*T*_*v*_	
Walking 1.0 m/s	RMSE (% L)	NRMSE (%)	RMSE (% L)	NRMSE (%)	RMSE (% BWL)	NRMSE (%)
Single-Task, Intra-Day	2.72	5.90	0.80	7.15	0.15	9.67
Single-Task, Inter-Day	5.25	10.89	1.21	12.54	0.30	20.08
Multi-Task, Intra-Day	2.76	6.21	0.85	6.77	0.18	11.25
	*F*_*ap*_		*F*_*v*_		*F*_*ml*_	
Walking 1.5 m/s	(% BW) RMSE	(%) NRMSE	(% BW) RMSE	(%) NRMSE	(% BW) RMSE	(%) NRMSE
Single-Task, Intra-Day	2.34	4.38	4.43	3.47	1.36	5.41
Single-Task, Inter-Day	6.19	11.76	14.59	11.36	2.61	10.24
Multi-Task, Intra-Day	2.53	4.74	5.37	4.19	1.44	5.69
	*C* *O* *P*_*ap*_		*C* *O* *P*_*ml*_		*T*_*v*_	
Walking 1.5 m/s	(% L) RMSE	(%) NRMSE	(% L) RMSE	(%) NRMSE	(% BWL) RMSE	(%) NRMSE
Single-Task, Intra-Day	3.14	5.25	0.79	6.60	0.20	9.35
Single-Task, Inter-Day	6.10	10.57	1.37	12.84	0.34	16.50
Multi-Task, Intra-Day	3.55	5.46	0.84	6.80	0.22	10.56
	*F*_*ap*_		*F*_*v*_		*F*_*ml*_	
Calf Raise	RMSE (% BW)	NRMSE (%)	RMSE (% BW)	NRMSE (%)	RMSE (% BW)	NRMSE (%)
Single-Task, Intra-Day	0.77	16.97	3.41	15.89	0.68	16.08
Single-Task, Inter-Day	1.39	32.46	7.58	39.63	1.45	34.92
Multi-Task, Intra-Day	1.57	37.22	5.33	24.98	0.91	22.48
	*C* *O* *P*_*ap*_		*C* *O* *P*_*ml*_		*T*_*v*_	
Calf Raise	RMSE (% L)	NRMSE (%)	RMSE (% L)	NRMSE (%)	RMSE (% BWL)	NRMSE (%)
Single-Task, Intra-Day	1.11	5.83	0.35	9.86	0.13	20.70
Single-Task, Inter-Day	3.78	20.29	0.89	28.06	0.25	44.59
Multi-Task, Intra-Day	1.78	9.19	0.49	14.32	0.13	21.74

Root mean squared (RMSE) and Normalized root mean squared error (NRMSE) for the six ground reaction force components of the walking at 1.5 m/s trial, and three select of the ground reaction kinetics of the calf raise task. The multi-task, intra-day model performs as well as the single-task, intra-day model which means that with sufficient training data, the insole can be used to study multiple locomotion tasks. Component Names: Anterior-Posterior Force (*F*_*ap*_), Vertical Force (*F*_*v*_), Medial-Lateral Force (*F*_*ml*_), Anterior-Posterior Center of Pressure (*C* *O* *P*_*ap*_), Medial-Lateral Center of Pressure (*C* *O* *P*_*ml*_), Vertical Torque (*T*_*v*_)

For walking at 1.0 m/s, the single-task, intra-day, the single-task, inter-day and the multi-task intra-day groups has normalized root mean square values (mean ± std) of 6.8 ± 2.4, 13.2 ± 3.5, and 7.0 ± 2.3, respectively. For walking at 1.5 m/s, the single-task, intra-day and single-task, inter-day had normalized root mean square error (NRMSE) values (mean ± std) of 5.7 ± 2.1 % and 12.2 ± 2.3 % respectively and the multi-task, multi-day group had a mean NRMSE value of 6.2 ± 2.3 %. For the calf raises, the single-task, intra-day and single-task, inter-day had NRMSE values (mean ± std) of 14.2 ± 5.4 % and 33.3 ± 8.6 % respectively and the multi-task, multi-day group had a mean NRMSE value of 21.7 ± 9.6 %.

***Ground reaction kinetics for walking at 1.0 m/s***

For walking at 1.0 m/s, the single-task, intra-day model had normalized root mean square error (NRMSE) values less than 10 % for all six components of the ground reaction kinetics (Table 2). The single-task, inter-day model had NRMSE values above 10 % for all six components of the ground reaction kinetics but the three ground reaction forces and the two center of pressure values have NRMSE values (10.9–13.1 %) close to the accuracy criterion. The multi-task, intra-day model has NRMSE values less than 10 % for all of the kinetic components with the exception of the vertical torque (*T*_*v*_).

***Ground reaction kinetics for walking at 1.5 m/s***

For walking at 1.5 m/s, the single-task, intra-day model had normalized root mean square error (NRMSE) values less than 10 % for all six components of the ground reaction kinetics (Table 2). The single-task, inter-day model had NRMSE values above 10 % for all six components of the ground reaction kinetics but the three ground reaction forces and the two center of pressure values have NRMSE values (10.2–12.8 %) close to the accuracy criterion. The multi-task, intra-day model has NRMSE values less than 10 % for all of the kinetic components with the exception of the vertical torque.

***Ground reaction kinetics for calf raises***

For the calf raise task, the single-task, intra-day model had normalized root mean square error (NRMSE) values less than 10 % for the anterior-posterior center of pressure and medial-lateral center of pressure positions (Table 2). None of the predictions of the single-task, inter-day model were less than 10 %. Only the prediction of the anterior-posterior center of pressure was less than 10 % for the multi-task, intra-day model.

### Ankle joint torque estimation

**Fig. 5:**
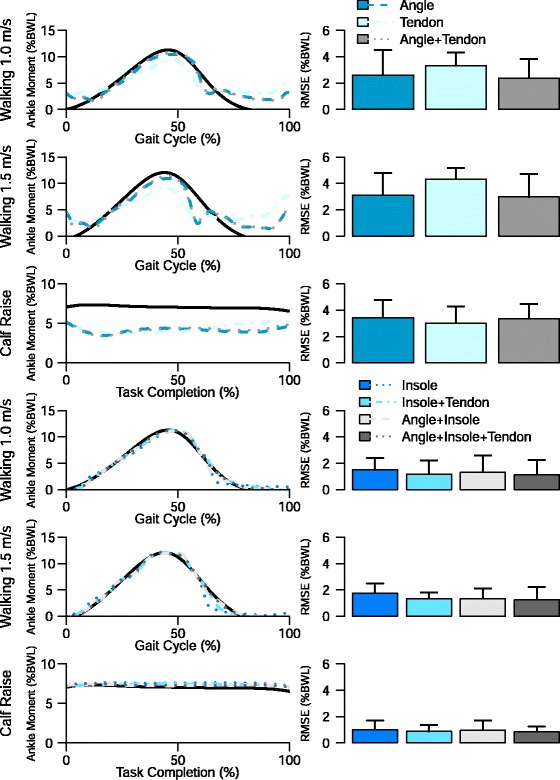
(*Top*) Mean normalized right ankle moments as calculated by Inverse Dynamics and estimated by the multi-task, multi-day model for each of the seven sensor combinations on the three tasks. The angle and tendon sensors are not sufficient to predict moment for all of the tasks by themselves. (*Bottom*) Normalized root mean squared error (NRMSE) for the seven sensor combinations on the three different tasks. The insole sensor alone outperforms the all of the other sensors individually. Combining the angle and tendon sensor with the insole has a small benefit for the NRMSE

Overall, the insole sensor individually was a stronger predictor of ankle joint torques than the tendon sensor or the ankle angle from inverse kinematics. In the multi-task, intra-day model, the insole sensor data was sufficient to predict the ankle joint torque for walking at 1.0 and 1.5 m/s tasks with normalized root mean square error (NRMSE) values of 8.7 and 9.2 % respectively (Table 3). The ankle angle and tendon features, both individually and together, had high normalized root mean square error (NRMSE) values and large deviations in the mean curves (Fig. 5) for all three tasks. Combining the insole data with the ankle angle data or the tendon data resulted in a lower NRMSE on all three tasks. For all tasks, the combination of insole and tendon data produced similar NRMSE as the combination of insole and ankle angle data.

**Table 3:** Fit metrics for the ankle moment estimation

Multi-task, Intra-day	Calf raise		Walking 1.0 m/s		Walking 1.5 m/s	
ankle joint torque	RMSE	NRMSE	RMSE	NRMSE	RMSE	NRMSE
features	(% BWL)	(%)	(% BWL)	(%)	(% BWL)	(%)
Angle	3.42	53.06	2.56	15.83	3.01	16.42
Tendon	3.01	46.02	3.33	20.47	4.34	23.54
Angle+Tendon	3.35	51.77	2.34	14.46	2.94	16.03
Insole	1.04	16.52	1.39	8.72	1.70	9.21
Insole+Tendon	0.90	13.78	1.17	7.42	1.30	7.04
Angle+Insole	0.98	15.57	1.27	8.04	1.36	7.29
Angle+Insole+Tendon	0.91	13.78	1.17	7.51	1.27	6.85

Root mean squared error (RMSE) and normalized root mean squared error (NRMSE) for the right ankle moment for the calf raise, walking at 1.0 m/s, and walking at 1.5 m/s trials. The angle and tendon sensors individually have low accuracy (NRMSE > 10 %) in all three tasks. The insole sensor has high accuracy for the walking tasks but not for the calf raise tasks. Combining redundant measurements from the insole sensor and the tendon sensor improves accuracy on all three tasks. Units: Body Weight * Leg Length (BWL)

## Discussion

Wearable sensors for estimating the ground reaction forces and the ankle joint moment can provide key biomechanical data for analyzing human motion. Our results show that a low-cost pressure insole and tendon sensor can produce estimates similar to the reported accuracy of commercial devices. For walking, a model produced by training both walking and calf raise data (multi-task, intra-day) produced normalized root mean square error (NRMSE) values for the ground reaction kinetics that were under 10 % for all three components of the force vector and both components of the location of the center of pressure (NRMSE: 4.15–6.80 %). The predictions of the vertical torque (NRMSE: 10.56 %, 11.25 %) exceeded the cutoff by a small amount. For the calf raises, only the prediction of the anterior-posterior center of pressure met the accuracy criterion. The results of the prediction of the AJT were similar to the GRF, where the sensor set had accuracy less than 10 % for walking but not for calf raises.

The results of the prototype sensors compare favorably with previous research on walking in commercial sensors [5, 6, 8]. Font et al. (2008) showed RMSE errors of 5 % for the vertical force, 12 % for the anterior-posterior force, and 28 % for the medial lateral force [6] for walking in the Pedar insoles. Rouhani et al. (2010) also found errors of 4 % for the vertical force, 7.4 % for the anterior-posterior force, 11.3 % for the medial-lateral force, and 14.7 % for the vertical torque in Pedar insoles. In a laboratory prototype, Howell et al. (2012) showed NRMSE of the ankle torque of 5.9 % for their healthy patients and 9.8 % in stroke subjects.

One advantage of the current insole prototype is that the pressure sensors respond to changes in the volume of the air bladder. The capacitive and resistive sensors found in commercial devices are uniaxial only but our prototype sensors produced a signal in response to three dimensional axial or sheer stress. The disadvantage is that the model between sensor voltage and a kinetic component like vertical force is no longer simply linear but our results show that the neural network training is sufficiently accurate.

Our study goes further than previous studies by evaluating our sensor system on a dataset that includes a non-stereotypical motion and multiple testing days. Despite the increased variability in the data set due to the inclusion of inter-day testing and data from both tasks, the multi-task, intra-day, and the single-task, intra-day models had similar accuracy which suggests that the current prototype may be limited by sensor accuracy and not task variability.

Recently Godi et al. (2014), suggested that plantar pressure insoles are potentially more accurate for spatial variables (like peak force and center of pressure) and worse for temporal variables like stance duration especially at lower sampling frequencies [25]. Our high accuracy for vertical force during walking suggests that our system could be used to accuracy predict contact time and stance duration.

For the ankle joint torque estimations, we found that generally the insole data was the more accurate than the ankle angle from inverse kinematics or the tendon sensor. The insole data alone were accurate for walking (RMSE ≤ 10 %) but insufficient for the calf raise task. Including, the tendon sensor, which had a lower individual NRMSE value than the insole sensor, lowered the NRMSE value for both the calf raise and the walking tasks. With both the insole and tendon sensor as features, the addition of the ankle angle calculated from inverse kinematics did not substantially improve the NRMSE value. The ankle joint torque predictions show the beneficial strategy of including sensor redundancy in estimation.

Our results demonstrate that estimating the calf raise task was particularly difficult for the prototype sensors. The plantar pressure insole predicted the majority of the ground reaction forces data for walking and but only very little for calf raises. One limitation of the prototype insole sensor is that the spatial resolution is low. During the calf raise task, the majority of the motion is spent balancing on the forefoot where only 5 of the 8 sensors are active which further decreases the spatial resolution of the signals. One way to improve the estimation algorithms across these dissimilar tasks would be to include knowledge of foot state in order to trigger different stored models.

Most subjects did not report any major comfort issues during the performance of the tasks. A few of the shorter subjects did complain that the tendon sensor was uncomfortable around the moment of peak calf raise height. An important consideration for at-home health monitoring systems is the burden of the system on the the likelihood of adoption and continued use by patients. Future work on the prototype sensors will focus on further reducing the weight and increasing the comfort of the sensors. Our prototype is also currently limited by the wired connection to the data acquisition system. However, the sensors all return voltage signals which means that an off-the-shelf microcontroller with an analog to digital converter component and a wireless emitter could readily be used to read in the sensor data.

Another point of future work will be to develop a proper set of stereotypical and non-stereotypical tasks to act as a calibration set for true at home monitoring. While calf-raises are an interesting balancing task, movements that are part of the activities of daily living such as reaching for items, turning and sit-to-stand transfers are important elements of future research.

## Conclusion

We developed a sensing system of two low-cost sensors for estimating the ground reaction forces and ankle joint torque of a data set which include walking at 1.0 m/s, walking at 1.5 m/s, and calf raises on multiple collection days. A multi-task, intra-day model was able to accurately predict the ground reaction forces and ankle joint torque at both walking speeds. The combination of the insole and the tendon sensor produced more accurate predictions than the individual sensors in both the walking and the calf raise tasks. Estimates of the ground reaction kinetics and ankle joint torque on the calf raise task were worse than walking which suggests task-specific deficiencies that should be further studied.
